# Biliary Atresia: A Case Report

**DOI:** 10.7759/cureus.75087

**Published:** 2024-12-04

**Authors:** Maliha Muzaffer, Anees Masarath, Fareedullah Mohammed

**Affiliations:** 1 Department of Pharmacy, Mesco College of Pharmacy, Hyderabad, IND

**Keywords:** biliary atresia, cholestatic liver disease, end-stage liver disease, extrahepatic biliary atresia, hepatobiliary iminodiacetic acid, histopathological examination, intraoperative cholangiogram, kasai portoenterostomy, liver transplantation, roux-en-y jejunojejunostomy

## Abstract

Biliary atresia (BA) is a serious hepatobiliary disorder that occurs due to progressive inflammation and scarring obstruction in the bile ducts, posing a threat to life. This condition usually appears in infants, and timely identification is fundamental for a better prognosis. If left untreated, individuals will inevitably experience liver damage and mortality. This case report describes a nine-month-old female infant presenting with jaundice, icteric sclera, yellowish skin, acholic feces, and hepatomegaly. Elevated liver enzymes and a hepatobiliary iminodiacetic acid (HIDA) scan confirmed BA. Histopathological examination revealed fibrosis, cholestatic disease, and an atretic gallbladder. A modified Kasai portoenterostomy (KPE) with Roux-en-Y jejunojejunostomy was performed, and the infant was discharged with supportive care. However, seven months post-Kasai portoenterostomy, the infant presented with persistent jaundice and progressive deterioration of liver function, indicative of a failed Kasai procedure.

Consequently, she was scheduled to undergo liver transplantation (LT) as a definitive treatment. BA is a rare disorder that is observed across nearly all ethnic groups, though the incidence rates vary significantly. This case highlights the efficacy of liver transplantation in treating failed Kasai procedures and demonstrates the potential for enhanced outcomes in infants with end-stage liver disease.

## Introduction

Biliary atresia (BA) is the primary surgically fixable liver issue in neonates, impacting approximately one in 8,000 (the Far East and Oceania) and 16,000 (Europe and North America) live-born infants with female predominance. The condition involves a total blockage of bile flow caused by the gradual destruction and closure of either a section or the entire external biliary system [1]. Additionally, the intrahepatic bile ducts are affected. It can present as two particular clinical phenotypes. Embryonic BA occurs in 10%-15% of cases and is characterized by early bile duct injury, often with the absence of extrahepatic bile ducts. Symptoms such as jaundice are present at birth. It is associated with genetic mutations and splenic malformations (8%-12%). Perinatal BA makes up 70%-80% of cases; this form typically occurs in infants who seem healthy at birth but develop cholestatic symptoms such as jaundice in the first weeks of life. It is linked to a type I cytokine response and can occasionally present with cardiovascular and intestinal malformations. Other forms include cystic BA (5%-10%) and BA associated with cytomegalovirus (CMV) (10%) [1]. The understanding of BA's pathophysiology remains unclear, and various theories exist about its development. BA involves immunological components, genetic susceptibility, environmental toxins, and viral infections [2]. The Japanese Society of Pediatric Surgery categorized biliary atresia into three types based on anatomical structure: type I (choledochal atresia), type II (common hepatic duct atresia), and type III (hepatic portal atresia). Type III BA was previously classified as the "incurable" type, with negative results impacting 80%-95% of patients [3].

Jaundice is the earliest sign of BA, typically appearing between birth and eight weeks, rarely later. Infants may have acholic stools and dark urine due to bilirubin excretion. As the disease progresses, a firm, enlarged liver and splenomegaly can develop. Early laboratory tests often reveal mild elevations in conjugated or direct bilirubin levels in presymptomatic infants later diagnosed with BA. An ultrasound can aid BA diagnosis by identifying features such as abnormal gallbladder size or shape, gallbladder contractility, the absence of the common bile duct, enlarged hepatic hilar lymph nodes, and the highly indicative "triangular cord" sign. The patency of the extrahepatic biliary tree can be evaluated using a hepatobiliary iminodiacetic acid (HIDA) scan. Histological features of BA consist of expanded portal tracts with bile duct proliferation, edema, fibrosis, inflammation, and bile plugs in the canaliculi and bile ducts. However, histology alone cannot differentiate BA from other obstructive causes. An intraoperative cholangiogram is considered the gold standard for diagnosing BA; if the contrast fails to fill the biliary tree or reach the intestine, a Kasai hepatoportoenterostomy (HPE) should be performed without delay [4]. This treatment aims to restore bile flow, reduce clinical jaundice, and maybe even reverse liver fibrosis, which might eventually save the liver. The procedure excises the completely obliterated extrahepatic biliary remnant, preserving the denuded (sometimes seemingly solid) portal plate, which is then anastomosed to a mobilized jejunal Roux loop. The results after Kasai portoenterostomy (KPE) can vary significantly in research and even more in practical situations with successful outcomes. Effective bile drainage occurs in approximately half of pediatric patients [5].

Post-KPE, medical care involves the supplementation of fat-soluble vitamins, ensuring adequate nutrition and avoiding potential complications. Liver transplantation (LT) is recommended for individuals who do not experience the complete resolution of jaundice or are impacted by severe complications, including portal hypertension, cholangitis, hepatopulmonary syndrome, and even malignancy due to the progression of liver injury [6].

## Case presentation

A nine-month-old female infant was brought to the emergency department by her mother with a high-grade, continuous fever for four days, accompanied by cough, cold, increased work of breathing for two days, and watery loose stools (2-4 episodes per day) for three days. The child had a prior history of hospitalization due to acute bronchiolitis. Upon examination during this admission, the infant appeared sick and febrile, with a heart rate of 120 beats per minute (bpm) and a respiratory rate of 54 breaths per minute. On respiratory system examination, bilateral crepitations were noted, and subcostal recession (SCR) was positive. The abdomen was soft but distended, and the infant weighed 5.5 kg.

Her medical history was notable for yellowish discoloration of the eyes and clay-colored stools since birth. At 29 days of age, she was admitted with these symptoms. Initial laboratory investigations, which included liver function tests (LFT), complete blood profile (CBP), coagulation profile, and serum calcium, revealed deranged liver parameters and anemia. In addition, serum electrolytes, blood urea, and creatinine were also performed, and all results were within the normal range (Table 1).

**Table 1:** Comparison of laboratory investigations at initial diagnosis and follow-up GGT, gamma-glutamyl transpeptidase; SGPT, serum glutamic-pyruvic transaminase; SGOT, serum glutamic-oxaloacetic transaminase; ALP, alkaline phosphatase; PT, prothrombin time; APTT, activated partial thromboplastin time; INR, international normalized ratio

Test	Results		Normal range
	At 29 days old	At nine months old	
Liver function tests			
Total bilirubin	9.18 mg/dL	16.59 mg/dL	<2 mg/dL
Direct bilirubin	5.9 mg/dL	8.33 mg/dL	<0.2 mg/dL
Indirect bilirubin	3.28 mg/dL	8.26 mg/dL	0.2-0.8 mg/dL
GGT	69 U/L	-	12-147 U/L
SGPT	64 U/L	215.2 U/L	12-45 U/L
SGOT	46 U/L	406.2 U/L	9-80 U/L
ALP	171 U/L	733.6 U/L	150-420 U/L
Biochemistry			
Calcium	-	7.7 mg/dL	8.8-10.8 mg/dL
Hematology			
Hemoglobin	9.5 g/dL	8 g/dL	10.5-14 g/dL
WBC	9.6 x 10^3^/mm^3^	9.1 x 10^3^/mm^3^	6-17 x 10^3^/mm^3^
Platelets	4.6 x 10^3^/mm^3^	1.6 x 10^3^/mm^3^	150-450 x 10^3^/mm^3^
Coagulation			
PT	11.5 seconds	15.1 seconds	11-15 seconds
APTT	40.5 seconds	39.6 seconds	30-40 seconds
INR	0.83	1.14	0.8-1.2

An abdominal ultrasound showed a partially distended gallbladder, and a hepatobiliary iminodiacetic acid (HIDA) scan confirmed biliary atresia. She subsequently underwent a modified Kasai portoenterostomy with Roux-en-Y jejunojejunostomy. Intraoperative findings included an atretic gallbladder, absent hepatic and cystic ducts, and cholestatic changes in the liver. Histopathological examination (HPE) of the liver revealed ballooning degeneration of hepatocytes, intrahepatic cholestasis with bile plugs, focal giant cell transformation, bile ductular proliferation, periportal fibrosis, and focal neutrophil infiltration, consistent with cholestatic disease and fibrosis. The gallbladder specimen showed markedly attenuated lining with congested vessels, hemorrhage, and fibrosis, suggestive of chronic cholecystitis. Based on these findings, a diagnosis of extrahepatic biliary atresia was confirmed.

Since then, the infant experienced recurrent symptoms, including pale stools, jaundice, and poor weight gain during follow-up visits. She also developed left-sided Bacillus Calmette-Guérin (BCG) lymphadenitis with pus formation following BCG vaccination. Needle aspiration was performed on two occasions, draining a total of 5-10 mL of pus. In addition, she required medical management for an incisional hernia.

During the current admission, a contrast-enhanced computed tomography (CECT) of the chest revealed patchy consolidation in the posterior segment of the right upper lobe and the superior and posterior basal segments of both lower lobes, indicative of an infective etiology. The laboratory investigations performed during the current hospital stay are detailed in Table 1. Additionally, serum electrolytes, urea, creatinine, and total serum protein were measured and found to be within the normal range. The patient was diagnosed with postoperative extrahepatic biliary atresia (EHBA) with portal hypertension and bronchopneumonia. Her hemoglobin level was 8 g/dL, which improved to 11.6 g/dL after a blood transfusion. She received oxygen therapy at 2 L/minute as part of her supportive care. Treatment included intravenous (IV) antibiotics-amoxicillin-clavulanate (300 mg twice daily for 10 days) and piperacillin-tazobactam (550 mg thrice daily for seven days), along with pantoprazole (6 mg once daily) for gastrointestinal protection. Symptomatic management consisted of paracetamol (5.5 mL IV four times daily) and saline nasal drops (four times daily). Nutritional support comprised vitamin A (5000 IU orally every alternate day), vitamin D3 drops (1 mL once daily), syrup Calcimax (a composition of calcium, magnesium, zinc, and vitamin D3) (2.5 mL once daily), and syrup multivitamin (2.1 mL once daily). For liver support, she was administered syrup ursodeoxycholic acid (1.2 mL three times daily) and syrup L-ornithine L-aspartate (2.5 mL twice daily). Additionally, vitamin E (200 mg orally every alternate day) was given as antioxidant therapy.

After a 22-day hospital stay, the infant's condition improved significantly. She became afebrile, her heart rate decreased to 118 bpm, respiratory sounds were equal bilaterally, and her respiratory rate decreased to 34 breaths per minute. The abdomen remained soft, and her discharge weight was approximately 5.3 kg.

The patient was discharged with the following medications: syrup vitamin D3 (2 mL; 400 IU) once daily, to support bone health and prevent rickets; capsule vitamin A (25,000 IU) once a month, important for vision, immune function, and skin health; capsule vitamin E (200 mg) once weekly, as an antioxidant to support cellular protection; syrup Udiliv (2 mL) three times daily, for liver support, particularly in managing cholestasis; syrup HepaMerz (1 mL) three times daily, to improve liver function and reduce bilirubin levels; syrup Tonoferon (1 mL) once daily, to address iron-deficiency anemia and support red blood cell production; and NasoClear drops (two drops) four times daily, for nasal congestion relief. Additionally, she was advised syrup paracetamol (3 mL) orally as needed, for pain and fever management; syrup ambroxol (2.5 mL) orally as needed, as a mucolytic agent to aid in respiratory clearance; and intramuscular vitamin K (5 mg) once a month, to prevent bleeding due to vitamin K deficiency.

Given the infant's ongoing medical challenges, particularly related to her liver condition, plans were made for a liver transplantation. She was referred to a government hospital for further follow-up and management.

## Discussion

BA is a serious condition in infants that can progress to cirrhosis, liver failure, and ultimately death if not treated. It is the leading cause of pediatric liver transplantation in developed countries, with an incidence of one in 17,000 live births in the United Kingdom, slightly higher in regions such as Japan and China (one in 8,000) [7]. There is a slight female predominance, particularly in syndromic forms. BA affects both intra- and extrahepatic bile ducts and is marked by cholangiolar plugging, proliferation, fibrosis, inflammatory infiltrates, and luminal obliteration in the extrahepatic bile ducts [7]. The cause of BA remains unclear, but it is believed to be multifactorial, with bile duct damage potentially arising from genetic, infectious, inflammatory, and/or toxic factors [8]. A history of consistently pale stools may indicate extrahepatic obstruction [9].

In symptomatic infants with biliary atresia (BA), laboratory results often show elevated direct or conjugated bilirubin, mildly raised aminotransferases, and a marked increase in gamma-glutamyl transpeptidase (GGT). Presymptomatic infants may exhibit mildly elevated conjugated bilirubin levels shortly after birth, which should prompt further monitoring. Early screening methods, such as measuring conjugated bilirubin levels or using stool color cards, have improved diagnosis and outcomes. The diagnosis of BA is confirmed through a combination of imaging, laboratory tests, and liver biopsy to rule out other causes of cholestasis. Infants must be evaluated promptly, as the success of surgical intervention decreases with increasing age at the time of surgery. The gold standard for diagnosing BA remains an intraoperative cholangiogram, often followed by the Kasai procedure if bile duct obstruction is confirmed [4]. Infants with biliary atresia (BA) typically present with jaundice, pale stools, and dark urine, despite normal meconium passage at birth. Some may have pigmented stools initially, gradually progressing to acholic stools. Without treatment, BA leads to end-stage liver disease and death within two years. The purpose of the Kasai procedure is to improve bile flow by eliminating the atretic portal plate and establishing a channel from the bile ductules to the intestines. Liver failure signs such as ascites and hypoalbuminemia are contraindications for KPE, leading to referral for liver transplantation (LT). Jaundice clearance is monitored, and growth failure could signal the need for LT [10]. Around 57% of cases attain a resolution of jaundice within six months. Postsurgical treatment includes intravenous antibiotics, followed by low-dose oral antibiotics for 8-12 weeks. Ursodeoxycholic acid is administered for at least 12 months to enhance the excretion of bile acids from the liver and reduce their intestinal reabsorption, thereby limiting their recirculation. Fat-soluble vitamin supplements (A, D, E, and K) are essential for preventing malnutrition, managing fat malabsorption, and reducing the effects of excessive metabolic breakdown. Medium-chain triglycerides may be provided for patients with steatorrhea. Steroid use has been controversial, with studies showing no significant benefit and potential risks, and immunomodulatory therapies remain debated as an adjunct to surgery [8,2].

Preventing biliary atresia (BA) remains challenging. Epidemiological studies suggest a potential association with maternal factors such as intestinal and genitourinary infections, the use of anti-inflammatory asthma medications during the first trimester, and substance abuse, which may increase the risk of developing the condition. However, there is currently no clinical evidence to confirm that addressing these risk factors can prevent the onset of BA in at-risk infants [11]. Research using human stem cells and organoids serves as a preclinical platform for disease modeling and drug testing, with potential for diagnostic and therapeutic advances. Stem cells could be used to modulate inflammation, immune dysregulation, or liver fibrosis, possibly as an adjunct to KPE, and tissue engineering may eventually replace liver transplantation. Collaboration between academia and industry could make these emerging treatments successful, potentially turning BA into a curable condition [11].

## Conclusions

BA is the most common cause of cholestatic jaundice in neonates, necessitating surgery. The primary treatment for BA is the Kasai operation, which is a surgical intervention designed to restore bile flow. It is usually done within the first 2-3 months of life, preferably before 60-90 days of age. If the Kasai surgery fails, the sole therapeutic choice for life-saving treatment is liver transplant. Liver transplantation restores liver function, reduces jaundice, and improves the overall quality of life. For successful results, prompt referral and surgical intervention are essential. End-stage liver disease can be definitively treated with liver transplantation, giving patients a second chance at life. Patients who receive proper care can live active and healthy lives following transplantation. Vigilant observation is necessary. Recent advances in imaging technology have enhanced the ability to predict BA during pregnancy, particularly through high-resolution maternal ultrasound scans. This case serves as a wake-up call to pediatricians to adopt appropriate intervention in early diagnosis, therefore saving infants' lives from potentially fatal illnesses.
